# Rapid detection of liver metastasis risk in colorectal cancer patients through blood test indicators

**DOI:** 10.3389/fonc.2024.1460136

**Published:** 2024-09-11

**Authors:** Zhou Yu, Gang Li, Wanxiu Xu

**Affiliations:** ^1^ Affiliated Jinhua Hospital, Zhejiang University School of Medicine, Jinhua, China; ^2^ College of Mathematical Medicine, Zhejiang Normal University, Jinhua, China; ^3^ Xingzhi College, Zhejiang Normal University, Jinhua, China

**Keywords:** colorectal cancer (CRC), colorectal cancer liver metastases (CRCLM), machine learning, blood test indicators, rapid detection

## Abstract

**Introduction:**

Colorectal cancer (CRC) is one of the most common malignancies, with liver metastasis being its most common form of metastasis. The diagnosis of colorectal cancer liver metastasis (CRCLM) mainly relies on imaging techniques and puncture biopsy techniques, but there is no simple and quick early diagnosisof CRCLM.

**Methods:**

This study aims to develop a method for rapidly detecting the risk of liver metastasis in CRC patients through blood test indicators based on machine learning (ML) techniques, thereby improving treatment outcomes. To achieve this, blood test indicators from 246 CRC patients and 256 CRCLM patients were collected and analyzed, including routine blood tests, liver function tests, electrolyte tests, renal function tests, glucose determination, cardiac enzyme profiles, blood lipids, and tumor markers. Six commonly used ML models were used for CRC and CRCLM classification and optimized by using a feature selection strategy.

**Results:**

The results showed that AdaBoost algorithm can achieve the highest accuracy of 89.3% among the six models, which improved to 91.1% after feature selection strategy, resulting with 20 key markers.

**Conclusions:**

The results demonstrate that the combination of machine learning techniques with blood markers is feasible and effective for the rapid diagnosis of CRCLM, significantly im-proving diagnostic ac-curacy and patient prognosis.

## 1 Introduction

Colorectal cancer (CRC) is a malignant tumor originating from the tissues of the colon or rectum. It is one of the common cancers globally, and considered as a serious threat to human health (1). In 2020, the newly diagnosed cases of CRC were 1.9 million, with 935,000 deaths attributed to the CRC, accounting for 9.4% of all cancer-related fatalities (2, 3). The worldwide occurrence of CRC has been steadily rising (4). CRC is the second most lethal cancer for both men and women, following lung cancer (5). At the time of initial diagnosis, approximately 20–30% of CRC patients have liver metastasis (LM) (6). This trend is expected to continue, primarily due to the changes in lifestyle and diet, population ageing, and genetic factors (7). Each year, millions of people worldwide are diagnosed with CRC, and millions lose their lives as a result (8). The development of CRC often remains covert, with many patients presenting no obvious symptoms in the early stages. Consequently, a considerable number of patients are identified at a late stage of diagnosis, leading to significant challenges in treatment and a high mortality rate. CRC exhibits invasiveness and tends to metastasize to surrounding tissues and organs, with colorectal cancer liver metastasis (CRCLM) being particularly common (9).

CRCLM refers to the spread of the CRC cells to the liver from the primary tumor site in the colon or rectum. It is a common occurrence in the advanced stages of CRC and significantly contributes to the prognosis and treatment outcome for the patient (10). One-fifth of the CRC patients were found to develop liver metastasis during the course of their disease (11–13). These are the factors that make the liver become the favorite target of metastasis: Anatomically, the liver is close to the colon and the rectum. A rich blood supply at the liver enhances the ability of the cancer cell to break and spread through the blood. The liver metastasis in CRC consists of multistep processes: the local invasion of cancer cells into blood vessels or lymphatic channels, circulate in the bloodstream, and colonization and growth in the liver parenchyma (14). After being established, it would lead to CRCLM. Further, the size and location of the liver metastases would then evoke a variety of symptoms, such as abdominal pain, fatigue, weight loss, and several others (15). Despite the improvement in treatment modalities, there still exists a lot of difficulty in managing liver metastasis that arises from CRC due to poor prognosis (16). Nevertheless, many advances in systemic therapies and multidisciplinary approaches to treatment have improved the survival outcome for some of these patients. In summary, CRCLM represents a significant difficult clinical challenge in CRC management. Early detection, accurate staging, and comprehensive treatment strategies of CRCLM are indispensable steps for optimizing patient outcomes and improving quality of life (17). It is important in formulating the strategy for effective treatment, prognostic assessment, direction of the monitoring of the patient for treatment, and providing precision treatment (18–20).

Early diagnosis of CRCLM, therefore, is very important. It forms an integral aspect of the management of the CRC patient and therefore needs high priority from physicians and patients (21). Timely detection of CRCLM makes it possible to take necessary treatment measures, which enhance the effectiveness of treatment and survival rates. The diagnosis of CRCLM gives a physician information about a patient’s prognosis. Diagnosis of CRCLM in determining the starting treatment plan and subsequently guiding monitoring and changes during treatment. It is of utmost importance for a physician to monitor the dynamics of the disease and efficacy of treatment on a routine basis through imaging examinations and monitoring of blood indicators, making changes if necessary to the treatment schedule to help the patient live as long and enjoy a good quality of life as possible (22). The definite diagnosis of CRCLM is usually made through the use of imaging techniques, including CT, MRI, or PET scans, which may demonstrate the presence, location, and size of liver lesions (23). Less sensitive imaging studies could result in false positive or negative results for small or early metastatic lesions. However, liver puncture biopsy technique is more invasive, and there is a high possibility of getting complications like bleeding and infection. It cannot adequately reflect the overall status of the tumor. The genetic testing is expensive: it involves highly specialized equipment, with high costs, and requires technical support, which may result in serious risks of misdiagnosis or missed diagnosis. These methods are common examination ways, but relatively, it is difficult to carry out the preliminary screening examination of CRCLM.

Blood test indicators, including liver function, renal function, electrolytes, cardiac enzyme profile, blood lipids, and tumor markers, are typically measured through blood tests. These indicators encompass various aspects of human physiology and pathology and are of significant importance for assessing organ function, diagnosing diseases, and monitoring treatment efficacy (24, 25). The blood sample is typically drawn and then sent for analysis, where healthcare professionals examine different components of the blood to obtain valuable information. Insights into a wide range of health indicators could be provided by blood tests (24, 26). They are used to diagnose and monitor various blood disorders, such as anemia, leukemia, and other abnormalities in the blood system (27). Low red blood cell counts and hemoglobin levels may indicate iron deficiency or other nutritional deficiencies. Recently, blood test indicators have also gained widespread attention in cancer detection (28–30).

Machine learning (ML) offers several beneficial applications for the diagnosis of diseases (8, 31–33), thereby showing its potential contribution in gaining high accuracy and efficiency for cancer diagnosis. An application based on ML models can be used for analyzing complex datasets to discover the patterns in diseases that exceed the level of intuitive judgments of human doctors (34). For example, in the diagnosis of lung cancer (35, 36), breast cancer (37–39), and many other types of cancers (40), the ML models have been experimentally proven to be equal to or even better than a radiologist in the diagnosis in some cases. A ML system can automate data analysis and, thus, the time between detection to diagnosis is shorter, especially for the diseases which need a rapid response like stroke and heart attack (41). One study published today reported findings in which ML has been helpful in identifying the most effective treatment plans for large groups of patients, offering data-driven support for personalized treatment. Such means would have medical treatment tailored to an individual’s specific prevailing situation. ML helps improve the accuracy of diagnosis with a better and efficient health outcome; hence, it reduces the threats related to medical misdiagnosis and overtreatment, which helps a person reduce undue cost associated with medical problems (42–44). ML models are designed to continue improving their diagnostic ability from new data.

In this study, the features were classified with different ML classifiers such as Adaptive Boosting (AdaBoost), Extremely Randomized Trees (ERT), Multi-layer Perceptron (MLP), Stochastic Gradient Descent (SGD), Random Forest (RF), EXtreme Gradient Boosting (XGBoost). The best optimal classifier selected in these classifiers of the features as blood markers for CRC and CRCLM. Additionally, considering that redundant features in the feature set could interfere with classification results and provide invalid information, the optimal feature subset was calculated by using a feature selection strategy. Specifically, p-values for each feature were computed to jointly select the most discriminative optimal feature subset, and then feature selection method was used to obtain the most optimal feature subset. Finally, the best classification accuracy for CRC and CRCLM classification was gained with the most optimal feature subset.

## 2 Materials and Methods

### 2.1 Datasets

The blood data were provided by Jinhua Central Hospital, which included 246 participants in CRC group, aged between 37 and 89, with 159 males and 87 females. There were also 254 participants in CRCLM group, aged between 23 and 91, with 174 males and 80 females. Participants in the CRC group were selected based on a confirmed diagnosis of pathological analysis of tissue samples. For the CRCLM group, participants were selected based on not only a confirmed diagnosis of CRC but also evidence of metastatic disease to the liver. This evidence could have been obtained through imaging test demonstrating liver lesions that were confirmed to be CRC metastases through additional testing of biopsy. This study also had exclusion criteria to ensure that participants did not have confounding medical conditions that could interfere with the results or place them at undue risk. These could include liver metastases caused by other primary tumors, as well as comorbid serious diseases, such as combined severe cardiovascular disease, organ failure, coagulation abnormalities and other cancers. The complete blood test in the data is shown in **Table 1**.

**Table 1:** Significant correlations with the risk of liver metastasis for the blood test indicators.

Item	Item Abbreviation	P value
White blood cell count	WBCC	5.2×10^-03^
Percentage of neutrophil	NP	5.9×10^-05^
Percentage of lymphocytes	POL	2.4×10^-08^
Percentage of eosinophils	POE	3.7×10^-01^
Percentage of monocytes	POM	1.1×10^-03^
Percentage of basophils	POB	7.4×10^-03^
Neutrophil count	NC	1.7×10^-04^
Monocyte count	MC	5.7×10^-06^
Basophil count	BC	2.5×10^-01^
Lymphocyte count	LC	1.4×10^-02^
Eosinophil count	ESC	3.1×10^-01^
Erythrocyte count	ETC	4.0×10^-02^
Haemoglobin concentration	HC	1.0×10^-02^
Specific volume of red blood cells	SVORBC	3.6×10^-03^
Mean hematocrit	MH	3.5×10^-02^
Mean red cell haemoglobin	MRCH	1.2×10^-01^
Mean erythrocyte haemoglobin concentration	MEHC	9.2×10^-01^
Erythrocyte volume distribution width	EVDW	3.2×10^-01^
Total blood platelet count	TBPC	1.2×10^-02^
Mean platelet volume	MPV	2.5×10^-01^
Hematocrit (blood platelet count)	BPC	1.7×10^-02^
Platelet volume distribution width	PVDW	8.5×10^-01^
Ultrasensitive C-reactive protein	UCP	4.8×10^-08^
Total protein	TP	2.1×10^-01^
Globulin	globulin	5.2×10^-01^
Albumin	albumin	3.7×10^-05^
Total bilirubin	TB	1.5×10^-02^
White ball ratio	WBR	3.8×10^-01^
Indirect bilirubin	IB	2.3×10^-03^
Direct bilirubin	DB	2.8×10^-01^
Glutamine aminotransferase, an amino acid	GATT	1.4×10^-02^
Glutamic transaminase, an amino acid	GST	9.1×10^-06^
Glutamate/glutamate	GTM	5.5×10^-04^
Y-glutamyltransferase	YG	4.3×10^-10^
Alkaline phosphatase	AP	2.4×10^-15^
Total bile acids	TBA	5.1×10^-02^
Potassium	potassium	2.8×10^-01^
Sodium	sodium	1.3×10^-03^
Chlorine	chlorine	1.2×10^-03^
Calcium	calcium	5.9×10^-08^
Phosphorus	phosphorus	1.7×10^-02^
Magnesium	magnesium	9.4×10^-03^
Creatinine	creatinine	9.0×10^-01^
Urea nitrogen	UN	6.2×10^-01^
Urea nitrogen: creatinine	UN:C	9.3×10^-01^
Uric acid	UA	6.2×10^-01^
Glucose	glucose	6.7×10^-01^
Lactate dehydrogenase	LD	1.2×10^-05^
Creatine kinase	CK	2.1×10^-01^
Creatine kinase isoenzyme	CKI	2.2×10^-05^
Myoglobin	myoglobin	4.0×10^-04^
Total cholesterol	TC	1.1×10^-02^
Triglyceride	triglyceride	5.0×10^-01^
Apolipoprotein A	AA	4.1×10^-01^
Apolipoprotein B	AB	1.3×10^-02^
High density lipoprotein	HDL	5.9×10^-03^
Low density lipoprotein	LDL	1.3×10^-03^
Lipoprotein (a)	L(a)	1.1×10^-02^
Alpha fetoprotein	AFP	1.9×10^-01^
Carcinoembryonic antigen	CEA	3.0×10^-04^
Glycoantigen 19-9	G 19-9	5.0×10^-10^
Prostate-specific antigen	PSA	4.7×10^-04^
Free prostate-specific antigen	FPSA	2.1×10^-01^
Free PSA: Total PSA	FP: TP	3.6×10^-01^
Glycoantigen 125	G125	2.7×10^-03^

### 2.2 ML classifier

#### 2.2.1 Adaptive boosting classifier

AdaBoost starts by training a weak classifier (typically a decision tree) on the original dataset. Once the first classifier is trained, AdaBoost increases the weights of the misclassified instances to focus more on the difficult cases. This process is repeated for a specified number of iterations or until perfect accuracy is achieved. Each classifier is assigned a performance-based weight. Finally, AdaBoost combines these weak classifiers into a single strong classifier by taking a weighted vote of their predictions (45). By focusing on difficult cases, AdaBoost minimizes errors on the training set and can achieve higher accuracy than individual classifiers. It can be combined with any learning algorithm, not requiring any parameter adjustments except for the number of iterations. Despite its iterative nature, AdaBoost can be less prone to overfitting compared to other powerful classifiers, especially if weak classifiers are simple. AdaBoost can select informative features, reducing the dimensionality and potentially improving the execution time.

#### 2.2.2 Random forest classifier

RF classifier is a powerful, versatile ML algorithm that constructs multiple decision trees at training time and outputs the class that is the mode of the classes of the individual trees. This is widely used for its robustness, simplicity, and effectiveness against all types of data. At the core of the RF algorithm lies an ensemble of decision trees trained on different subsets of the dataset. This is done using a technique called “Bagging”, by repeatedly sampling observation data points from the dataset with replacements to train each tree (46–48). An additional randomness is incorporated while building each tree, which is the selection of a random subset of features at each split in RF. This ensures that diversity is achieved among the trees, strengthening performance and decreasing the risk of overfitting. Once all the trees are trained, we make predictions by aggregating the predictions of all trees. The trees are used to predict outputs, and most frequently, the predicted label by trees is used to cast a vote similar to a majority voting system in classification tasks. Unlike individual decision trees, which can easily overfit to the training data, the aggregation method in RF helps in minimizing overfitting, making it robust even when dealing with complex datasets. RF can achieve high accuracy in many tasks, outperforming many other classifiers, including some more complex algorithms. It can handle large datasets with high dimensionality and does not require feature scaling. RF can provide estimates of feature importance, which can be very informative in understanding which features are contributing most to the decision-making process. It is effective for classification and requires very little tuning of parameters.

#### 2.2.3 Extremely randomized trees classifier

ERT classifier is an ensemble learning technique similar to RF, designed to further randomize tree building in the quest for model simplicity and variation. Due to the random nature of feature and split selection, ERT can be much faster to train than more traditional algorithms like RF, particularly on large datasets (49, 50). By averaging multiple trees, it can reduce the variance and help prevent overfitting, similar to other ensemble methods. ERT’ method of using random thresholds for each feature makes it less sensitive to noise in the input data. Moreover, its fewer parameters to tune as compared with other algorithms, such as Gradient Boosting or RF, undoubtedly for the choice of split point at each node. Similarly, just like the RF, an ERT model can give some idea of the importance that it has in some data within the training data and hence be quite helpful in the feature selection process.

#### 2.2.4 Multi-layer perceptron classifier

MLP classifier is a type of neural network classifier used to estimate tasks where an approximate function maps input features to discrete output classes. MLP is a deep, artificial neural network containing more than one perceptron. It consists of an input layer, multiple hidden layers, and an output layer (51, 52). MLP is capable of modeling the complex nonlinear relationship between input and output. MLP can be designed with varying the number of layers of an MLP and the neurons to meet the variance in complexities presented by the data. MLP classifiers are capable of handling noisy input data. It is quite useful in classification tasks, equipped with adaptations to handle multiple output decisions. MLP can be trained with large datasets and scaled by increasing the number of hidden layers or neurons to further increase its ability to model very complex relationships. However, MLPs are still haunted by several weaknesses, such as prone to overfitting, especially very deep networks, or in the presence of inadequate training data. All that aside, MLPs require plenty of computational resources while in the middle of the training process, especially about changing the network architecture.

#### 2.2.5 Stochastic gradient descent classifier

SGD classifier is a general optimization procedure, using many objective functions. It can be used with many objective functions, even in some cases replacing various ML algorithms for training linear classifiers and neural networks. It’s particularly favored for large-scale and online ML problems (53–55). In contrast to the conventional Gradient Descent method, where the model parameters are updated based on the gradient computed across the entire dataset, SGD updates these parameters using the gradient of the loss function concerning a randomly selected individual sample. It’s much faster than computing the exact gradient because it uses only one data point (or a small batch) at a time, which significantly reduces the computational burden when dealing with large datasets. For large datasets, it can converge faster to the minimum, as each step of the learning process is applied immediately, allowing the model to start improving right away. SGD is well-suited for online learning models where new data continuously flows in because it can update the model incrementally, without the need for retraining from scratch. It can be used with a variety of loss functions and models, making it adaptable to classification tasks. The inherent randomness in its updates can help the algorithm to escape local minima, potentially leading to better solutions in some cases.

#### 2.2.6 Exreme gradient boosting classifier

XGBoost is efficiency, flexibility, and portability (56, 57). The crucial difference is the use of regularization to control overfitting and hence being effective even for non-smooth optimizations. XGBoost is designed to be highly efficient and can run more than ten times faster than other gradient boosting techniques. A number of optimization techniques is utilized, including efficient handling of sparse data and parallel processing. With default settings, XGBoost gives better results, even outperforming other tools, owing to its powerful handling of a variety of data types, relationships, and distributions. This is a built-in support for the measurement of feature importance and can be very useful when trying to figure out which features had the most impact for the predictions. It handles a plethora of diverse objective functions and evaluation criteria for classification tasks.

### 2.3 Feature selection

This study first subjected the dataset to an initial feature selection process, utilizing the one-way analysis of variance (ANOVA) method. In this phase, p-values were calculated for each feature, with a threshold of 0.05 set to determine statistical significance. Features with p-values less than or equal to 0.05 were deemed significant, indicating a potential relationship with the response variable for further investigation.

Next, the AdaBoost algorithm, a powerful ensemble learning method, was employed to analyze the importance of the selected features. AdaBoost not only evaluated the significance of each feature but also ranked them in descending order based on their influence in classification problems. This step produced a ranked list of feature importance, serving as a robust guide for subsequent feature selection.

Adopting a stepwise approach, the study then iteratively analyzed the feature importance array. At each iteration, the top “n” features (where “n” corresponded to the current iteration number) were extracted and used to construct classification models. These models were trained and tested to measure their classification accuracy, which served as the criterion for evaluating the quality of the feature subsets.

By systematically adding features, the study observed how the classification accuracy changed as the number of features increased. Throughout this process, detailed records were maintained, focusing on feature sets that notably improved model accuracy. When no further gains in accuracy were observed or a decline commenced, the optimal feature subset was identified. This subset encompassed the most crucial features contributing significantly to enhancing model accuracy.

The highest classification accuracy achieved was associated with the specific combination of features within the optimal subset. This not only facilitated model interpretability but also improved the model’s generalization capability on unseen data. The hybrid feature selection strategy, combining statistical significance and ML algorithm evaluation, efficiently reduced the feature space, enhancing computational efficiency while ensuring the predictive performance of the model remained intact. In handling high-dimensional data, this approach offers a highly viable and efficient solution.

### 2.4 Evaluation metrics for classifiers

As for this research, classification accuracy was measured using five-fold cross-validation, of which it ranks one of the most popular methods to find the performance of ML models. It consists in splitting the dataset into five equal parts and training the model five times, each time with four folds as a training set and one fold as a validation set. After each training iteration, you validate the model using the validation set, typically with some performance metric such as accuracy, precision, recall, etc. Finally, averaging was done between the results of the five training iterations, and standard deviation for the final result was calculated. In addition, during the five-fold cross-validation, the random introduction of noise that may not be representative of the population into the model is reduced by repeating training and validation many times over, thus giving the estimate of model generalization greater accuracy and making it easier to detect if issues of overfitting or underfitting plague the model.

This study evaluates the performance of the model using accuracy, precision, recall, and F1 score. Here are the definitions of accuracy, precision, recall, and F1 score based on true positives (TP), false negatives (FN), true negatives (TN), and false positives (FP). Accuracy is the proportion of correct predictions (both TP and TN) among the total number of predictions made by the model. Precision is the proportion of TP among all the instances that the model predicted as positive. It measures how accurate the model is in identifying positive instances. Recall is the proportion of TP that the model correctly identified out of all the actual positive instances. It measures how well the model is able to find all the positive instances. F1 Score is the harmonic mean of precision and recall. It provides a single score that balances the trade-off between precision and recall. An F1 Score reaches its best value at 1 and worst score at 0. TP, FN, TN, and FP were obtained by comparing predicted labels with true labels. Specifically, TP represents the number of positive labels predicted correctly as positive, FN represents the number of positive labels predicted incorrectly as negative, FP represents the number of negative labels predicted incorrectly as positive, and TN represents the number of negative labels predicted correctly as negative.

### 2.5 Statistical analysis

All statistical analyses were performed using SPSS version 26.0 (IBM Corp., Armonk, NY, USA). Continuous variables were expressed as mean ± standard deviation (SD). Continuous variables were expressed as mean ± standard deviation (SD). All statistical tests were two-sided, and p-values less than 0.05 were considered statistically significant.

## 3 Results

### 3.1 Classification effects of classifiers

**Table 2** presents the performance metrics for six different ML models in predicting the diagnosis of CRC and CRCLM through cross-validation. In the comparative analysis of ML models, AdaBoost exhibited the highest precision at 89.4%, demonstrating the strongest ability to distinguish between CRC and CRCLM diagnoses. It also achieved the highest predictive accuracy of 89.3%, indicating it as the most reliable model for correct predictions in testing. Both the F1 score and recall rate evaluation metrics showed the best performance, both at 89.3%. Although the RF model did not outperform AdaBoost, it still demonstrated relatively high accuracy, precision, F1 score, and recall rate, reaching 89.3%, 89.2%, 89.3%, and 89.3%, respectively. The overall performance of MLP was the lowest, with accuracy, precision, F1 score, and recall rate all at their lowest values. This suggests that they may not be as effective, which could pose significant implications for medical diagnosis if CRCLM is not identified, leading to adverse consequences. The superior performance of AdaBoost can be attributed to its adoption of the strategy of adaptive weight adjustment, whereby higher weights are assigned to misclassified samples, thereby focusing the model more on difficult-to-classify samples and improving its ability to classify boundary samples. In conclusion, AdaBoost emerges as the most promising model for predicting CRC and CRCLM based on routine blood test indicators, providing a particularly robust approach.

**Table 2:** Classification effects of multiple ML models.

Classifier	Accuracy (%) (mean ± sd)	Precision (%) (mean ± sd)	F1 Score (%) (mean ± sd)	Recall (%) (mean ± sd)
ERT	84.2 ± 3.5	84.7 ± 3.1	84.1 ± 3.6	84.2 ± 3.5
MLP	79.6 ± 1.9	80.1 ± 1.7	79.4 ± 2.1	796 ± 1.9
SGD	80.4 ± 4.3	83.0 ± 2.0	80.0 ± 4.9	80.4 ± 4.3
RF	89.3 ± 5.0	89.2 ± 5.0	89.3 ± 5.0	89.3 ± 5.0
XGBoost	86.7 ± 4.2	86.8 ± 4.3	86.7 ± 4.2	86.7 ± 4.2
AdaBoost	89.3 ± 3.5	89.4 ± 3.5	89.3 ± 3.5	89.3 ± 3.5

### 3.2 Optimization results of feature selection

In this study, a preliminary statistical testing was conducted to screen a subset of features with p-values less than 0.05, demonstrating significant correlations with the target variable, as shown in **Table 1**. Subsequently, the importance of these features was further analyzed using the AdaBoost classifier, yielding a global ranking of feature importance. This step was crucial as it helped identify the features that exerted the greatest impact on the model’s predictive ability. Following the acquisition of the feature importance ranking, the top n-ranked features were selected in an attempt to achieve optimal classification performance. The selection of the number of features, denoted as n, was based on multiple iterations and adjustments through experimentation.

**Figure 1:**
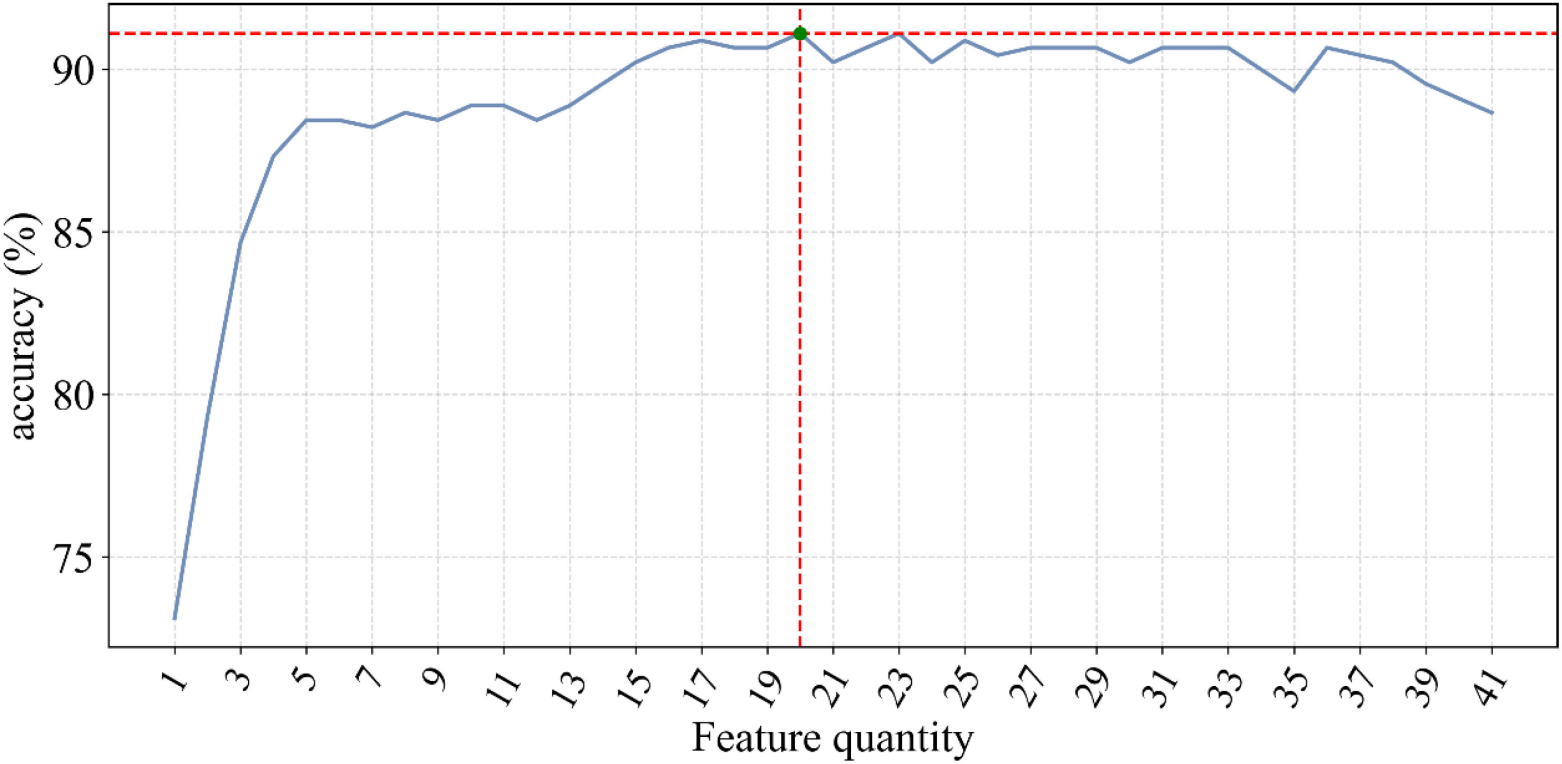
Relationship between number of ranked features and accuracy.

The fluctuation of classification accuracy with the variation of n was meticulously recorded, as depicted in **Figure 1**. From this graph, it is evident that as n increases, the classification accuracy gradually improves until reaching a peak, after which further increases in n result in decreased accuracy. This indicates that a greater number of features does not always lead to better model performance but rather suggests an optimal number of features. The feature importance scores from the Adaboost model, are displayed in **Table 3**. These scores are calculated. In this case, when the number of features reached 20, the classification accuracy peaked at 91.1 ± 4.6%.

**Table 3:** Feature importance ranking.

Feature Name	Feature Importance	Accuracy (mean ± sd)
CKI	1	73.1 ± 1.6
G125	2	79.3 ± 2.7
CEA	3	84.7 ± 4.5
G 19-9	4	87.3 ± 3.0
AP	5	88.4 ± 3.7
YG	6	88.4 ± 4.8
LD	7	88.2 ± 4.6
PSA	8	88.7 ± 3.3
UCP	9	88.4 ± 5.1
myoglobin	10	88.9 ± 4.6
calcium	11	88.9 ± 4.4
GST	12	88.4 ± 4.4
sodium	13	88.9 ± 4.9
IB	14	89.6 ± 4.1
HDL	15	90.2 ± 4.5
L(a)	16	90.7 ± 4.9
POL	17	90.9 ± 4.2
AB	18	90.7 ± 4.8
NP	19	90.7 ± 3.9
TB	20	91.1 ± 4.6
magnesium	21	90.2 ± 4.9
chlorine	22	90.7 ± 4.4
GTM	23	91.1 ± 4.5
ESC	24	90.2 ± 4.9
MC	25	90.9 ± 4.3
albumin	26	90.4 ± 5.0
phosphorus	27	90.7 ± 4.2
POM	28	90.7 ± 4.8
LC	29	90.7 ± 4.8
MH	30	90.2 ± 5.5
NC	31	90.7 ± 5.1
LDL	32	90.7 ± 5.0
WBCC	33	90.7 ± 4.7
GATT	34	90.0 ± 4.2
HC	35	89.3 ± 4.7
SVORBC	36	90.7 ± 5.3
ETC	37	90.4 ± 5.5
TBPC	38	90.2 ± 4.1
BPC	39	89.6 ± 5.2
TC	40	89.1 ± 4.5
POB	41	88.7 ± 3.7

This significant improvement in performance compared to the initial classification is detailed in **Table 4**. Such optimization notably enhances the predictive capability of the model while mitigating the risk of overfitting potentially induced by feature redundancy.

**Table 4:** Comparison of classification effects after feature selection.

Feature Type	Accuracy (%) (mean ± sd)	Precision (%) (mean ± sd)	F1-Score (%) (mean ± sd)	Recall (%) (mean ± sd)
All Features	89.3 ± 3.5	89.4 ± 3.5	89.3 ± 3.5	89.3 ± 3.5
Optimal Features	91.1 ± 4.6	91.1 ± 4.6	91.1 ± 4.6	91.1 ± 4.6

**Figure 2:**
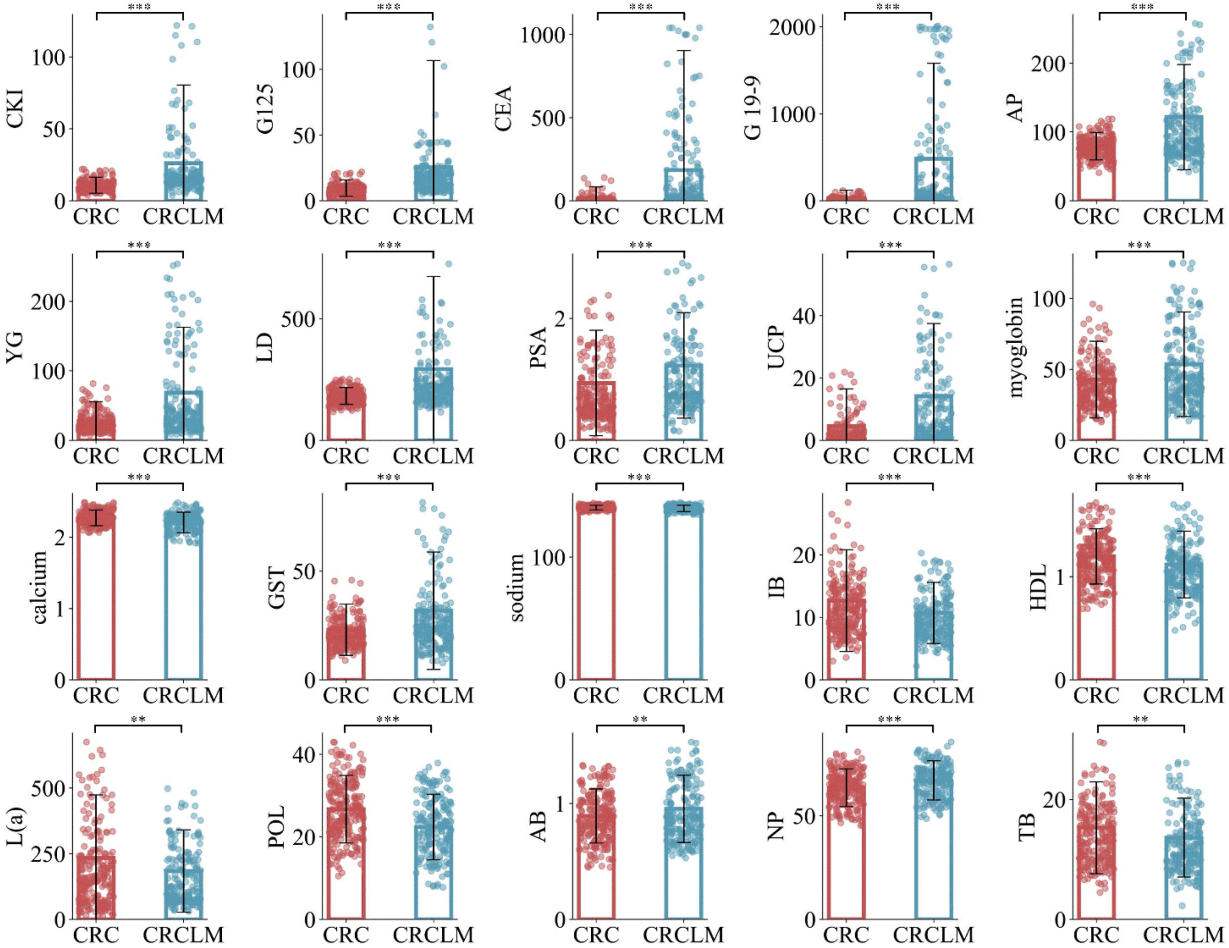
Mean values of the comparison between CRC and CRCLM in the optimal feature subset. ** means p ≤ 0.01, and *** means p ≤ 0.001.

Furthermore, to gain a deeper understanding of the biological significance of these 20 optimal features between CRC and CRCLM, detailed statistical analyses were conducted. Specifically, we computed the mean expression levels of these features in CRC and CRCLM, followed by a comparative analysis. Additionally, to validate the statistical significance of these differences, relevant p-values were calculated. Visualization of these data is presented in **Figure 2**. The results indicate that the expression differences of the majority of features between the two groups are statistically significant, further emphasizing the potential crucial roles of these features may play in the development and metastasis of CRC.

Through this series of analyses and experiments, the accuracy of the classification model was improved and a deeper understanding of the biomarkers associated with CRC and CRCLM was gained. This discovery would make a more efficient biological basis for the early diagnosis and targeted treatment of CRC and CRCLM. Additionally, such a methodological approach demonstrates the feasibility in performing effective feature selection and classification modeling for complex biomedical data, and in doing so, provides robust technical support and a theoretical foundation for future related research efforts.

## 4 Discussion

The current CRCLM diagnosis has faced several challenges, from early detection to accurate diagnosis. It is in this study that ML models are used for the diagnosis of CRCLM, basing on blood test indicators. Some promising results were found in these studies. The derived results of this research show possible value in using ML models for CRCLM diagnosis. Notably, in the classification of CRC and CRCLM, the AdaBoost model depicted the best discriminative performance, carrying out excellent predictive accuracy. This means that the combination of blood test markers and ML algorithms could be applied to develop a working model for the early prediction of CRCLM. The present study findings bring out the performance differential of various ML models in the diagnosis of CRCLM. By using ML algorithms and blood test indicators, it is possible to create a fast and accurate way for the early diagnosis of metastases of CRC to the liver and thus make a tool useful even in general clinical practice to improve treatment and patient care processes. The findings of the study thus contribute to a novel approach in the diagnostic determination of CRCLM and are therefore valuable for further research, from basic sciences to clinical applications of medical diagnostics.

The most important twenty contributing factors (ranked as per importance) in CRCLM diagnosis, according to our Adaboost model, are: CKI, G125, CEA, G19-9, AP, YG, LD, PSA, UCP, myoglobin, calcium, GST, sodium, IB, HDL, L(a), POL, AB, NP, TB. In this study, it is observed that CKI, G125 and CEA can achieve 85% classification accuracy. In patients with liver metastasis, elevated levels of certain CKI have been observed. This elevation may be due to increased cellular turnover and damage caused by the metastasis of cancer cells to the liver. Multiple studies have reported the correlation between elevated CKI and liver metastasis in CRC patients. These studies emphasize the potential of CKI as non-invasive biomarkers for monitoring disease progression and metastasis. CEA is one of the most commonly used tumor markers for CRC (4, 58). CA125 is mainly used as a marker for ovarian cancer, but its levels may also be elevated in some CRC patients, especially those with CRCLM. In clinical practice, a single tumor marker, such as CEA, to differentiate between CRC and CRCLM is not very specific. However, the combination of CKI, G125, and CEA will have good clinical significance and value. Further attention could be paid to these indicators in future studies.

In CRCLM patients, myoglobin levels may be elevated. This elevation could be due to increased tumor burden leading to muscle damage or tissue destruction caused by liver metastasis. When the tumor invades the liver and causes liver cell damage, myoglobin may be released into the blood as a non-specific response. In colorectal cancer patients, particularly those with liver metastasis, HDL levels are often significantly elevated. This elevation may be due to the high metabolic activity of tumor cells and tissue damage caused by liver metastasis. Rapid tumor cell proliferation and high metabolic activity lead to increased lactate production, which is further exacerbated by liver metastasis. CA19-9 is primarily used for the detection of pancreatic cancer. Although PSA is mainly used for the detection of prostate cancer, its potential association with CRCLM cannot be ignored. The elevation of CA125, CA19-9, and PSA suggests the possibility of malignancy or metastasis. Monitoring these tumor markers is of significant value for diagnosis, prognosis evaluation, and treatment monitoring. Liver lesions, including liver metastasis, can lead to elevated levels of AP. Elevated AP suggests liver involvement or bone metastasis. YG levels are elevated in liver diseases and in patients with liver metastasis. The detection of YG helps assess liver function and metastasis. GST plays a crucial role in detoxification, and liver damage or liver metastasis can cause changes in its levels. The detection of GST is used to assess liver function and metastasis. Liver diseases and liver metastasis can lead to elevated levels of IB. Elevated IB indicates liver function damage or bile duct obstruction. The detection of TB is used to assess liver function and metabolic health, indicating the importance of liver function blood tests in assessing the risk of liver metastasis. The detection of NP is used to rule out cardiovascular system issues and assess liver function. Total cholesterol levels may change in patients with liver dysfunction and metastasis. When tumor cells are metabolically active, POL levels may rise, especially during metastasis. The detection of POL is used to assess tumor activity and metastasis. Elevated levels of TB are common in liver diseases and metastasis. UCP levels are elevated in liver lesions and patients with liver dysfunction. The detection of UCP helps assess liver function and metastasis. Liver metastasis can cause abnormal calcium metabolism, leading to elevated or decreased blood calcium levels. Abnormal calcium levels indicate liver function abnormalities or bone metastasis. Liver dysfunction and metastasis can lead to electrolyte imbalances, including abnormal sodium levels. The detection of sodium levels is used to assess liver function and electrolyte balance. HDL levels may change in patients with liver disease and metastasis. The detection of HDL is used to assess cardiovascular risk and liver function. L(a) levels are associated with the risk of various cancers and may change during liver metastasis. The detection of L(a) is used to assess cardiovascular disease risk and tumor progression. The detection of AB is used to assess liver function and bile duct health.

In this regard, the findings provide a new perspective regarding the significance level in the use of hospital routine blood indicators for the diagnosis of CRCLM. At a larger scope that includes the twenty factors identified by the Adaboost model, the factors identify the blood test indicators accounted for by the diagnosis of CRCLM (1, 4, 7, 59). These results are consistent with established literature, where these factors have previously been considered potential risk factors for the diagnosis of CRCLM. Undoubtedly, the Adaboost model accurately identifies key driving factors for the diagnosis of CRCLM, marking the formulation of targeted intervention strategies to improve the initial critical stages of CRCLM. Adaboost models are becoming increasingly important in oncology, providing multifaceted approaches (60–63). This powerful algorithm has been proven particularly valuable (64, 65). The major applications where Adaboost has contributed majorly are the prediction of the risk of cancer, survival, diagnosing the staging, and assisting in classifying the cancer. The abilities of Adaboost models to easily aggregate different sources of data, hence identifying complex patterns, have transformed the advance understanding of cancer in improving patient outcomes.

This study provides valuable insights into the rapid detection of CRCLM from CRC, but it is important to acknowledge several limitations that may affect the interpretability and generalizability of our results. Firstly, the sample size in this study is relatively small and primarily focused on patients from a specific region, which may limit the generalizability of the results. Therefore, future research needs to validate the findings in larger and more diverse CRC populations to ensure broader applicability. Secondly, while this study demonstrates the potential of rapidly detecting CRCLM patients from CRC patients through blood test indicators, its limitations and the challenges of clinical implementation should not be overlooked. Training healthcare professionals is essential when introducing this detection method into clinical practice. Additionally, seamlessly integrating this approach into existing diagnostic workflows without increasing the clinical burden is another significant challenge. Future research should focus on expanding sample sizes, validating and standardizing detection methods, assessing cost-effectiveness, and developing practical clinical integration strategies. By addressing these issues, this method can be further advanced for clinical application, ultimately improving patient outcomes.

## 5 Conclusions

Blood testing indicators can increase the likelihood of diagnosis for CRCLM in community hospitals. This study focused on utilizing traditional ML algorithms and a feature selection strategy, to investigate the classification of CRCLM and CRC. In terms of CRCLM, AdaBoost algorithm achieved high accuracy reaching a maximum of 89.3%. The results of this algorithm were applied to identify the top 20 most relevant blood test features. Following feature selection strategy, the accuracy increased to 91.1% in the classification study. The findings of this study indicate that the AdaBoost model combined with blood testing indicators can be used to identify the liver metastases from CRC. Based on the above analytical framework and conclusions, the practical implication of this study is that it provides a potential tool for clinicians to rapidly identify patients who may have CRCLM, which can be integrated into existing diagnostic workflows and lead to earlier detection and treatment. In the future, this study will explore larger and more diverse datasets to confirm the findings of this study, and develop testing systems for clinical use.

## Data Availability

The data analyzed in this study is subject to the following licenses/restrictions: Implementing measures to protect the data from unauthorized access, disclosure, alteration, or destruction. Requests to access these datasets should be directed to yu-zhou.2008@163.com.
